# Global patterns of nitrate storage in the vadose zone

**DOI:** 10.1038/s41467-017-01321-w

**Published:** 2017-11-10

**Authors:** M. J. Ascott, D. C. Gooddy, L. Wang, M. E. Stuart, M. A. Lewis, R. S. Ward, A. M. Binley

**Affiliations:** 1 British Geological Survey, Maclean Building, Crowmarsh, Oxfordshire OX10 8BB UK; 2 British Geological Survey, Environmental Science Centre, Nicker Hill, Keyworth, Nottinghamshire NG1 5GG UK; 3 Lancaster Environment Centre, Lancaster University, Lancaster, LA1 4YQ UK

## Abstract

Global-scale nitrogen budgets developed to quantify anthropogenic impacts on the nitrogen cycle do not explicitly consider nitrate stored in the vadose zone. Here we show that the vadose zone is an important store of nitrate that should be considered in future budgets for effective policymaking. Using estimates of groundwater depth and nitrate leaching for 1900–2000, we quantify the peak global storage of nitrate in the vadose zone as 605–1814 Teragrams (Tg). Estimates of nitrate storage are validated using basin-scale and national-scale estimates and observed groundwater nitrate data. Nitrate storage per unit area is greatest in North America, China and Europe where there are thick vadose zones and extensive historical agriculture. In these areas, long travel times in the vadose zone may delay the impact of changes in agricultural practices on groundwater quality. We argue that in these areas use of conventional nitrogen budget approaches is inappropriate.

## Abstract (web summary)

Current global-scale nitrogen (N) budgets quantifying anthropogenic impacts on the N cycle do not explicitly consider nitrate storage in the vadose zone. Here, using estimates of depth to groundwater and nitrate leaching between 1900–2000, the authors show that the vadose zone is an important store of nitrate.

## Introduction

It is estimated that inputs of reactive nitrogen (N) into the terrestrial biosphere are currently more than double pre-industrial levels due to modern agricultural practices and application of N fertilisers^1^. Reactive nitrogen cascades through the environment and has resulted in deterioration in quality of groundwater and surface water used for public supply^2^ and ecological degradation of freshwater and marine systems^3^. To manage the impacts of additional reactive nitrogen, N budgets have been developed at a wide range of scales to quantify man’s impact on the N cycle^1,4,5^. These budgets typically assume a steady state over a 1 year timescale, with no net accumulation of N. However, recent work at both national and catchment scales has shown this to be inappropriate, as there can be substantial (and increasing) storage of nitrate in soils, the vadose zone and groundwater^6–9^. The slow travel time for solutes through the vadose zone means that significant amounts of dissolved reactive N may be stored. This also results in a significant lag between any changes in agricultural practices to reduce nitrogen loadings and subsequent impacts on groundwater and surface water quality^10^. Although the problems associated with time lag and storage of nitrate in the vadose zone have been identified at local^11–18^, regional^19–21^ and national scales^9,10,22–24^, the global significance of these processes has not yet been quantified. In this study, we hypothesised that long travel times in the vadose zone make it an important store of nitrate not considered at a global scale to date.

We quantified the nitrate stored in the vadose zone globally by linking numerical models and published datasets of nitrate leaching^25^, depth to groundwater^26^, recharge rate and porosity^27^ (see ‘Methods’ section). We considered the sensitivity of model outputs to changes in model inputs by varying nitrate leaching inputs, vadose zone effective saturation and travel time. Results are aggregated by lithology and basins, and analysed using the *k*-means cluster analysis^28^. The model was validated by comparing the model storage against previous national and catchment scale vadose zone storage estimates^6,9^ and by comparing model nitrate concentrations in recharge at the water table with observed concentrations in Europe^29^ and the USA^30^. We show that the vadose zone is an important store of nitrate at the global scale, with significant storage in areas with extensive historical agricultural development and large depths to groundwater. Use of conventional N budgets in these areas is likely to be highly limited and policymakers should consider vadose zone nitrate storage when planning pollution mitigation measures.

## Results

### Global spatiotemporal distribution of vadose zone nitrate

**Fig. 1:**
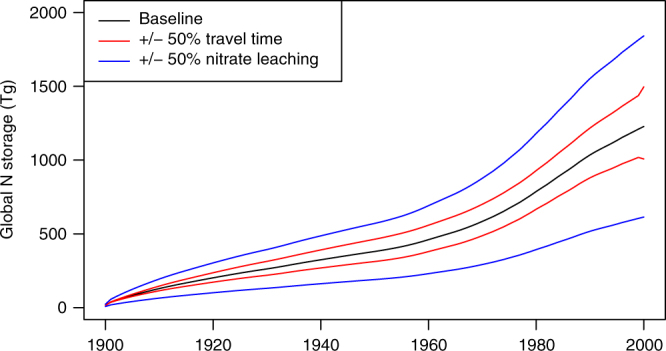
Modelled global increase in nitrate stored in the vadose zone between 1900 and 2000. Nitrate storage (in Tg N) is modelled under the baseline model run (black) and from sensitivity analyses (red and blue for +/− 50% travel time and nitrate leaching, respectively

**Fig. 2:**
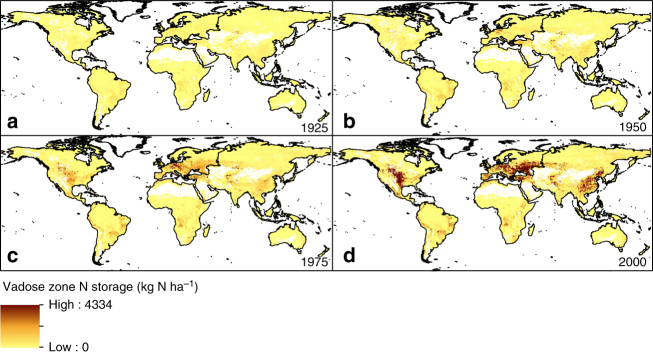
Spatial distribution of nitrate stored in the vadose zone. Global vadose zone N storage (in kg N ha^−1^) is shown for 1925 (**a**), 1950 (**b**), 1975 (**c**) and 2000 (**d**)

Our modelling shows a substantial continuous increase in the amount of nitrate stored in the vadose zone (Fig. 1). This implies the steady state assumption adopted by conventional nutrient budgets is not appropriate at relatively short timescales (<50 years). On the basis of the sensitivity analysis, for the year 2000, we estimate the total global storage to be between 605 and 1814 Tg N (Fig. 1). The range of values of nitrate storage associated with uncertainty in nitrate leaching inputs (605–1814 Tg N) is significantly greater than that for uncertainty in unsaturated zone travel time (1007–1496 Tg N) or vadose zone saturation (778–1227 Tg N). Modelled estimates of nitrate stored in carbonate vadose zones are estimated to be 9.6% (58–174 Tg) of total N storage. In these areas rapid transport may occur and observed storage may be limited due to low matrix porosity, and consequently model estimates are likely to be overestimates. Total vadose zone N storage is small (<3%) in comparison to estimates of total soil nitrogen (68,000^31^–280,000^32^Tg N), but potentially significant (7–200%) in comparison to estimates of more labile soil inorganic nitrogen (NO_3_^−^ + NH_4_^+^, 940^31^–25,000^32^Tg). The modelled spatiotemporal distribution of nitrate stored in the vadose zone (Fig. 2) shows substantial increases between 1950 and 2000 associated with increased global use of N fertilizers and subsequent leaching. Basins in North America, China and Central and Eastern Europe have developed large amounts of nitrate stored in the vadose zone due to thick vadose zones, slow travel times and high nitrate loadings.

Comparisons between estimates of nitrate storage made in this study with previous works go some way to validating the modelling undertaken. Previous studies have derived the amount of nitrate stored in the vadose zone for the Thames Basin^6,9^, England and for the countries of England and Wales and the USA^9^. The calculated peak store of 0.059 Tg N for the Thames catchment in this study agrees broadly with the range of peak nitrate storage values reported in previous work in this area (0.016–0.24 Tg N). For England and Wales, we calculated a peak store of 1.7 Tg N, which agrees with previous calculations, estimating the store to be 0.8–1.75 Tg N. For the USA, a first estimate of 29 Tg N was previously made^9^ and our modelling suggests a store of 191 Tg N. This large discrepancy can be accounted for by the modelling approach of the previous study, which only considered land areas where agriculture was >40% of the overall land use.

**Fig. 3:**
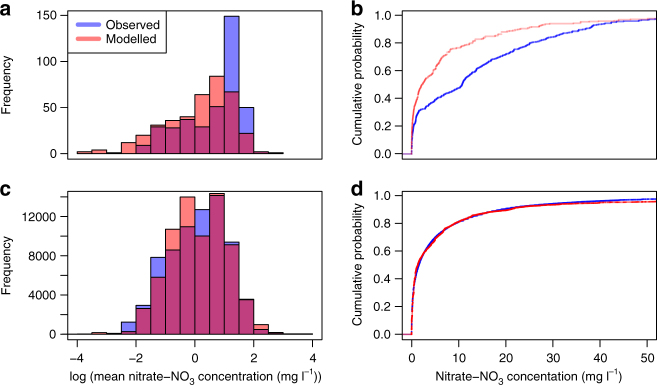
Observed and modelled distributions of nitrate concentrations in groundwater. Distributions of observed (blue) nitrate concentrations in groundwater and modelled (red) nitrate concentrations in recharge at the water table for the European Union^29^ (**a**, **b**) and the United States^30^ (**c**, **d**). Purple colour in the histogram indicates where the model and observed concentration distributions overlap

The distributions of observed groundwater nitrate concentrations and modelled concentrations in groundwater recharge show reasonably good agreement for both European Union and United States (Fig. 3). It should be noted that comparison between observed groundwater concentrations and concentrations in recharge do not take into account dilution of recharge by low nitrate groundwater. Consequently, comparison between these datasets should be considered to be a sense-check, but nonetheless useful, validation. The distributions of nitrate concentrations in the USA appear to be closer which reflects the much larger observational dataset for the USA than for Europe (see ‘Methods’).

### Coherent basin-scale nitrate storage trends

**Fig. 4:**
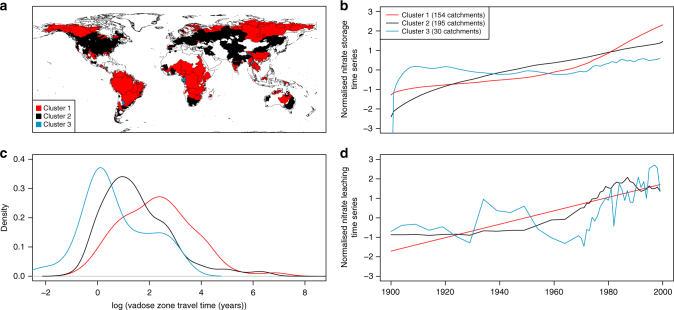
Basin-scale nitrate storage trends. Spatial distribution of the nitrate storage clusters (**a**), nitrate storage cluster centroids (**b**), distribution of vadose zone travel times (**c**) and mean annual nitrate leaching input time series (**d**) for each cluster

*k*-means cluster analysis revealed three spatially coherent responses in basin nitrate storage (Fig. 4a, b) reflecting differences in vadose zone travel time (Fig. 4c) and nitrate leaching inputs (Fig. 4d). In all the clusters, the time taken for the impact of stopping N leaching inputs from the base of the soil zone (ie, *N*_leach_ = 0, see ‘Methods’) to reach groundwater (*N*_out_ = 0) will equal the vadose zone travel time. The majority of basins fall within clusters 1 and 2. These clusters show a continuous increase in the nitrate stored in the vadose zone. The vadose zones in basins in these clusters accumulate nitrate with no loss to groundwater as the travel time through the vadose zone is long (Fig. 4c) due to deep water tables and low recharge rates. In these catchments, some legacy nitrate may not have reached the water table yet and anticipated improvements in groundwater and surface water quality due to catchment management may be significantly delayed. It should also be noted that there may also be significant lags in the saturated zone between recharge at the water table and discharge at receptors such as public water supply wells and streams, where there are long groundwater flow paths. In addition, in some areas where groundwater recharge is estimated to be very low, modelled estimates of vadose zone nitrate are likely to represent storage in both the soil and vadose zone.

Cluster 3 shows a substantially different nitrate storage response to the other clusters. This is a result of shorter vadose zone travel times. In these basins, storage rapidly increases initially until the travel time is reached and nitrate is present across the full depth of the vadose zone. After this point, the basin moves to a quasi-steady state where any input of nitrate from the base of the soil zone is accompanied by an equivalent loss from the base of the vadose zone to groundwater. This dynamic balance results in minimal increases in nitrate storage and a relatively rapid response to changes in N loadings in comparison to other clusters. In these catchments, nitrate loss at the base of the soil zone >10 years ago is likely to now be present in groundwater.

The nitrate leaching time series for each cluster (Fig. 4d) show distinct differences associated with the extent of historical agricultural and population development. Cluster 1 shows a continuous increase in nitrate leaching inputs through time associated with increased development and intensification of agricultural to maximise crop yields. Basins in cluster 1 form a spatially coherent pattern, covering large parts of the developing world including Africa, Southeast Asia and South America. Cluster 2 shows an increase in nitrate leaching to c. 1985, followed by decreases to 2000. Such an input can be characterised by historical agricultural development followed by implementation of catchment measures to reduce nitrate losses in the last decade. Spatially this cluster reflects large parts of the developed world including the USA and Europe. The nitrate leaching time series for cluster 3 shows significant variability associated with the small number of catchments averaged but generally shows an increase to 2000. Recent studies have shown evidence that nitrogen losses from agriculture follow an Environmental Kutznets Curve (EKC), with a number of developed countries having reduced nitrogen losses since the 1980s associated with increased GDP^33^. The spatiotemporal patterns of nitrate leaching inputs between the different clusters (Fig. 4d) corroborate this.

## Discussion

There is a well-established discourse on the balance between increasing agricultural productivity to improve human health and feed growing populations and the negative impact of nitrogen leaching on aquatic ecosystems^5^. A central tenet of future nitrogen management is that agricultural productivity can be increased in a cost-effective manner with limited environmental impacts through increased nitrogen use efficiency (NUE) and reduced soil nitrogen surplus (*N*_sur_)^33,34^. Several recent studies have continued to assume that *N*_sur_ is directly analogous to nitrate pollution^33,35,36^ and recently developed models that do consider groundwater explicitly still ignore the vadose zone^25^. Given the substantial lags present in the transport of nitrate from the soil zone to groundwater and surface water, we argue that use of *N*_sur_ alone as a metric to quantify impacts of agriculture on the aquatic environment is inappropriate. Our modelling shows that the vadose zone is a globally significant store of nitrate, which needs to be considered in future N budgets for more effective long-term nutrient management. N storage in the vadose zone is most significant in areas where agricultural development and intensification occurred first and where there is a large depth to groundwater. Storage of nitrate in the vadose zone is one of a number of temporary catchment retention processes such as storage in soil organic matter^8^, subsoils, land not in agricultural production^7^, the riparian zone and in rivers^6,37^. These possible nitrogen stores and how they change through time (eg, N release through mineralisation of soil organic matter) should also be compared with storage in the vadose zone to determine whether they are significant enough to be incorporated into future nutrient budgets. In combination, these processes will result in substantial delays in the impacts of changes in agricultural management practices on groundwater and surface water quality.

Nitrate storage in the vadose zone has significant implications for environmental policy. The need for internationally cooperative policy responses to nitrogen pollution to avoid shifting of pollution sources to areas with less stringent environmental controls has been established^38^. However, development of such policies is in its infancy^36^. Moreover, established policies in the developed world have been shown to be difficult to implement in areas where vadose zone lags are present. For example, it is now widely acknowledged^22,39^ that original environmental targets set under the European Water Framework Directive^40^ and Nitrates Directive^41^ may not be met due to storage of nitrate in the vadose zone. As a result, many river basin managers have been forced to consider new planning timescales accounting for these lags^22^.

Recent work^37^ has called for the development of integrated pollution management policies which consider both pollution sources and temporary (eg, vadose zone lags) and permanent (eg, denitrification) retention processes at the basin scale. Our work presented here provides a critical contribution to the literature in that we make the first global-scale quantification of one of these temporary processes. The spatial distribution of vadose zone N storage in 2000 (Fig. 2) can give a first global indication to policymakers and decision makers of where N legacy issues may be significant and delay improvements in groundwater and surface water quality. In these areas, an understanding of nitrate storage in the vadose zone can help managers in planning mitigation measures and the timescales and expectations for improvements in water quality. With this quantification of vadose zone N storage and further research to quantify other retention processes at the global scale, development of integrated pollution management strategies at an international level should be possible. Such an approach is critical for a realistic assessment of environmental impacts of global nitrogen flows associated with economic development and international trade^36^.

The spatial coherence of the nitrate storage clusters (Fig. 4) highlights the need for different management strategies to tackle nitrate pollution across developing and developed countries. In the developed world, a number of countries are already on a trajectory of declining soil N losses associated with sustainable intensification of agriculture^33^. In the developing world, soil N losses are increasing associated with rapid early development of fertilised agriculture^33^. However, in both cases, catchment retention processes such as vadose zone storage must be considered. Without consideration of these lags, which is often the case, nitrate pollution control policy may appear not to be working. This may lead to more stringent but unnecessary measures that may adversely impact agricultural production and/or lead to disproportionate costs.

## Methods

### Estimates of vadose zone travel time

Travel time in the vadose zone was derived by estimating the depth to groundwater and nitrate velocity. Depth to groundwater mapping at 0.5° scale was derived from previously published global groundwater model forced by modern climate, terrain and sea level^26^. Velocity of nitrate (${V_{{\rm{N}}{{\rm{O}}_3}}}$, m year^−1^) in the vadose zone was calculated as follows:

1 $${V_{{\rm{N}}{{\rm{O}}_3}}} = \frac{R}{\emptyset },$$

where *R* is the recharge rate (m year^−1^) and ∅ is effective porosity (dimensionless). Global groundwater recharge mapping was derived from the PCR-GLOBWB model^42^, which has been used extensively in global-scale hydrological modelling^43–45^. PCR-GLOBWB calculates vertical water fluxes between two soil layers and groundwater based on unsaturated hydraulic conductivity estimates for each layer^46^. Unsaturated hydraulic conductivity was calculated using the degree of saturation of each layer. This was calculated based on average, saturated and residual soil moisture content, in turn derived by depth of water storage in each layer and the layer thickness. Global soil mapping^47^ and soil moisture characteristic curves^48^ were used to derive soil physical relationships for each layer, tabulated moisture retention, matric potential and unsaturated hydraulic conductivity values.

Although recharge estimates derived using PCR-GLOBWB account for increased hydraulic conductivity with increased saturation, vadose zone velocities can also decrease with increased saturation associated with an increased cross-sectional area of flow^49^. On the basis of previous catchment and regional scale approaches^22,49–51^, we accounted for this process separately from recharge in the calculation of deep vadose zone travel times. Estimates of travel time through the deep vadose zone calculated using Eq. 1 assumes a fully saturated matrix. This is supported by work that shows that vadose zone velocities calculated using this method agree well with observed velocities derived from vadose zone porewater profiles in limestone and sandstone aquifers^10^. However, in partially saturated media, assuming 100% effective saturation will result in unsaturated zone velocities being underestimated and hence vadose zone storage being overestimated. N storage in vadose zones of strongly karstified aquifers with limited matrix porosity will also be overestimated using this method. Global geological maps do not differentiate between karst and non-karst sedimentary carbonate rocks^52^, so we explored the impact of these assumptions on model results through sensitivity analysis (see below).

### Estimates of nitrate leaching from the base of the soil zone

Nitrate leaching (*N*_leach_, kg N 0.5° grid cell^−1 ^year^−1^, same units for all N budget terms) at the base of the soil zone was derived from the global nutrient model IMAGE^25^ for 1900 to 2000. IMAGE has been detailed extensively elsewhere^4,25,53^ and the key soil zone N inputs, outputs and processes are described here for clarity and illustrated in Supplementary Fig. 1. IMAGE uses the concept of an annual steady state soil N budget surplus, defined as the balance between soil N inputs and outputs for a unit land area. Storage and release of N associated with changes in soil organic matter through time are not considered. Historic land cover data^54^ at the 0.5° scale, which distinguishes between 9 agricultural land use types and 17 different natural ecosystems was used as a basis to derive 5 broad land use groups for the soil N budget estimation (Supplementary Fig. 1). The soil N budget (*N*_budget_) is calculated as follows:

2 $${N_{{\rm{budget}}}} = {N_{{\rm{fix}}}} + {N_{{\rm{dep}}}} + {N_{{\rm{fert}}}} + {N_{{\rm{man}}}} - {N_{{\rm{withdr}}}} - {N_{{\rm{vol}}}},$$

where *N*_fix_ is biological nitrogen fixation, *N*_dep_ is atmospheric N deposition, *N*_fert_ is application of N fertilizer, *N*_man_ is addition of manure and *N*_withdr_ and *N*_vol_ are loss terms for N withdrawal from harvesting and ammonia volatilisation, respectively.

Biological nitrogen fixation in leguminous (pulses and soybeans) crops and natural ecosystems was estimated by crop production data and N content^4,55^. It was assumed that total biomass of leguminous crops was twice that of the harvested product, and that N is also released to the soil during the growing season^53^. Fixed N is available for harvesting, or volatilisation and leaching if released to the soil. Total N fixation during the growing season was therefore derived by multiplying the N in harvested product by three to account for this additional unharvested biomass and the plant–soil N flux^53^. Atmospheric N deposition for the year 2000 was estimated from an ensemble of global atmospheric chemistry models^56^ and estimated for 1900 to 2000 by scaling the N deposition field with historic emissions inventories^4^. Country level N fertilizer application rates divided by land use for 1900 to 2000 were derived from global databases^55,57^ and data on fixed N use in 1913^58^. Country animal population data in conjunction with N excretion rate estimates^59^ were used to estimate addition of N in manure form. Animal populations back to 1900 were derived from statistical compilations by Mitchell^60–62^ and scaling of human population data^63^ for poultry and camels where data was limited. N loss through ammonia volatilisation was estimated using a empirical model of c. 1700 field measurements across a range of different crop types, fertilizer types and applications and environmental conditions^64^. Removal of N through harvesting was estimated from country crop production data, crop dry matter and N content estimates^65^. N budget inputs and outputs derived from crop type and production data (*N*_fix_, *N*_man_, *N*_withdr_, *N*_vol_) were estimated back to 1900 by scaling 1960 crop production data with population numbers and land use data in the HYDE database^66^.

It is assumed that all reduced N compounds are nitrified to nitrate such that *N*_budget_ = soil nitrate^53^. When *N*_budget_ is positive, leaching, surface runoff and denitrification can occur. N leaching (*N*_leach_) at the base of the soil zone is a fraction of the soil N budget excluding N loss via surface runoff (*N*_sro_):

3 $${N_{{\rm{leach}}}} = {f_{{\rm{leach}}}}\left( {{N_{{\rm{budget}}}} - {N_{{\rm{sro}}}}} \right),$$

where the soil leaching fraction, *f*_leach_, is complementary to the fraction of soil N lost by denitrification (*f*_den_):

4 $${f_{{\rm{den}}}} = 1 - {f_{{\rm{leach}}}},$$

*f*_leach_ is estimated empirically using five denitrification factors, each with a range from 0 to 1, with a maximum value of 1:

5 $${f_{{\rm{leach}}}} = \left[ {1 - {{\rm MIN}}\left[ {\left( {{f_{{\rm{climate}}}} + {f_{{\rm{text}}}} + {f_{{\rm{drain}}}} + {f_{{\rm{soc}}}}} \right),1} \right]} \right]{f_{{\rm{landuse}}}},$$

where *f*_climate_ *, f*_text_ *, f*_drain_ *, f*_soc_ and *f*_landuse_ are factors representing climate, soil texture, aeration, soil organic carbon content and land use, respectively^25^. *f*_climate_ uses the Arrhenius equation and estimates of soil water capacity and potential recharge to estimate the effects of temperature and residence time on root zone denitrification^25^. *f*_text_ *, f*_drain_ *, f*_soc_ were estimated using global-scale mapping of soil texture, drainage and organic carbon content^53,67^. *f*_landuse_ was set to 1 for arable land areas, with grassland and natural vegetation having a value of 0.36^68^. For further detail on soil N budget inputs, outputs and processes the reader is referred to previous modelling studies^4,53^.

### Calculation of nitrate storage in the vadose zone

Nitrate storage in the vadose zone was calculated using a simple summation approach. It was assumed that nitrate undertakes conservative transport in the vadose zone. This is supported by numerous studies^69^, which showed that the evidence for vadose zone denitrification is very limited, with just 1–2% of the nitrate leached from the soil zone removed^70^. In some specific local hydrogeological environments (eg, where anaerobic conditions and organic carbon are present^69^), vadose zone denitrification may occur, and in these areas the model may overestimate nitrate storage. However, at the global scale this was considered negligible. For a year *t* (years), the nitrate stored in vadose zone, *N*_VZ_ (Tg N) for a grid cell with a vadose travel time, TT_VZ_ (year) and a time variant nitrate leaching input, *N*_leach_ (kg N), can be calculated as:

6 $${N_{{\rm{VZ}}}} = \frac{{\mathop {\sum }\nolimits_{i = t - {\rm{T}}{{\rm{T}}_{{\rm{VZ}}}}}^t {N_{{\rm{leach}}}}}}{{{{10}^9}}}.$$

Global maps of the model input datasets and the derived vadose zone storage for the year 2000 are shown in Supplementary Fig. 2. We derive changes in nitrate storage in the vadose zone through time using a simple mass balance approach;

7 $${N_{{\rm{leac}}{{\rm{h}}_t}}} - {N_{{\rm{ou}}{{\rm{t}}_t}}} = \Delta {N_{{\rm{VZ}}}},$$

where *N*_out_ (kg N) is the nitrate flux from the unsaturated zone to the saturated zone and$\Delta {N_{{\rm{VZ}}}}$ (kg N) is the change in nitrate storage in the vadose zone.

### Sensitivity and cluster analysis

We undertook a heuristic sensitivity analysis by running the model using different inputs. We separately varied the vadose zone travel time and nitrate leaching input by +/−50%. We also varied vadose zone effective saturations (0.25, 0.5, 0.75 and 1) to account for variable cross-sectional area of flow in partially saturated media.

We aggregated vadose zone N storage data by lithology and catchments. We separated areas underlain by sedimentary carbonate rocks^27^ to account for rapid vadose zone transport in karstic aquifers with limited matrix porosity, and hence limited N storage. We normalised the catchment nitrate storage responses for 1900–2000 and used *k*-means clustering^28^ to identify spatial patterns of N storage responses. Two, three and four clusters were tested and three gave the most coherent spatial pattern. For each of the clusters, we calculated the mean annual nitrate-leaching input for 1900–2000 and the kernel density distribution of travel times for the catchments within the cluster.

### Model validation

We undertook a two step model validation: (1) comparison against previously published national and catchment scale estimates of nitrate storage and (2) comparison against nitrate concentrations in groundwater. Recent work has given estimates of nitrate storage for the United Kingdom and the USA^9^ and for the Thames catchment^6^, England. We estimated nitrate concentrations in recharge at the water table as follows:

8 $${\rm{Conc}} = \frac{{{N_{{\rm{out}}}}}}{{{\rm{Recharge}}}}.$$

Modelled estimates of nitrate concentrations in recharge were compared against observed groundwater nitrate data for Europe^29^ and America^30^. It should be noted that this comparison does not directly validate estimates of nitrate storage. Comparison against observed nitrate concentrations in groundwater provides a sense-check that the nitrate inputs and vadose zone travel time estimates are reasonable.

### Data availability

Global input datasets (depth to groundwater table, recharge rate, porosity and nitrate leaching) and model validation data (groundwater nitrate concentrations) are publically available from the references cited in the ‘Methods’ section. Vadose zone nitrate storage data generated during the current study are available from the corresponding author on request.

## Electronic supplementary material

Supplementary Information
