# Supplementary material for: Global patterns of nitrate storage in the vadose zone
Source: Nat Commun. 2017 Nov 10;8:1416. doi: 10.1038/s41467-017-01321-w (PMC5680259; doi:10.1038/s41467-017-01321-w)
Supplement: Supplementary file 1 — Supplementary Information [file 41467_2017_1321_MOESM1_ESM.pdf]

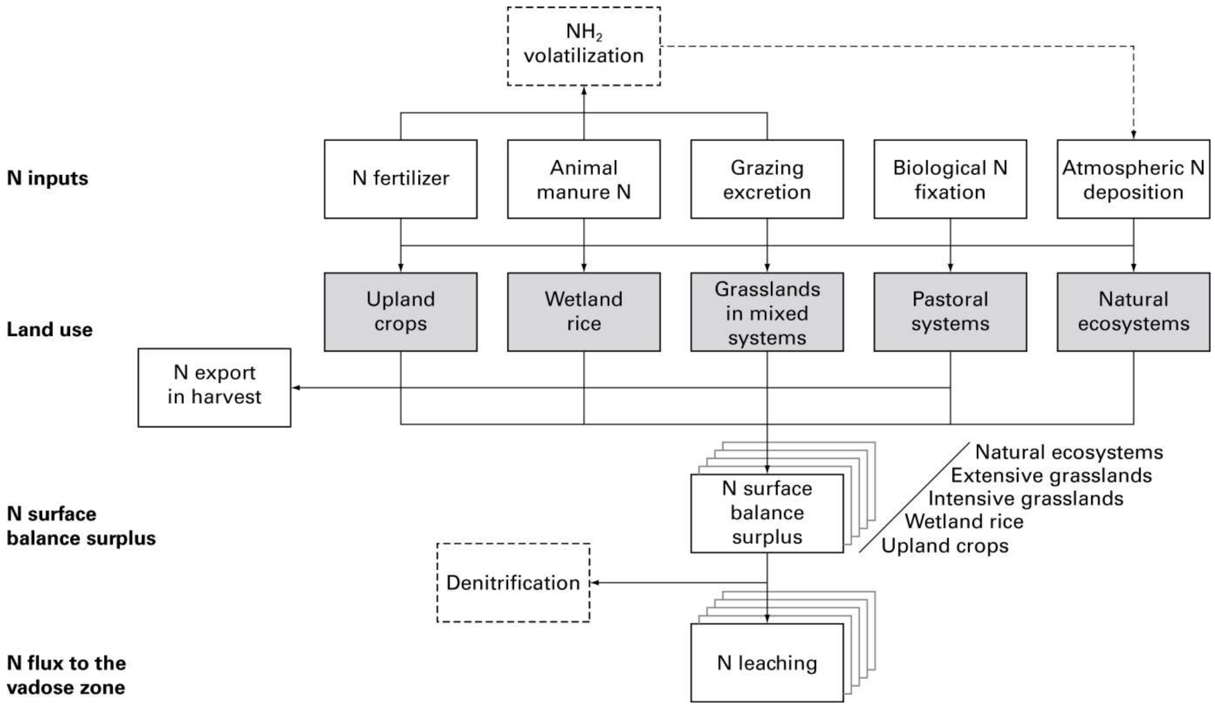

Supplementary Fig. 1: Scheme used to calculate N leaching at the base of the soil zone[1]

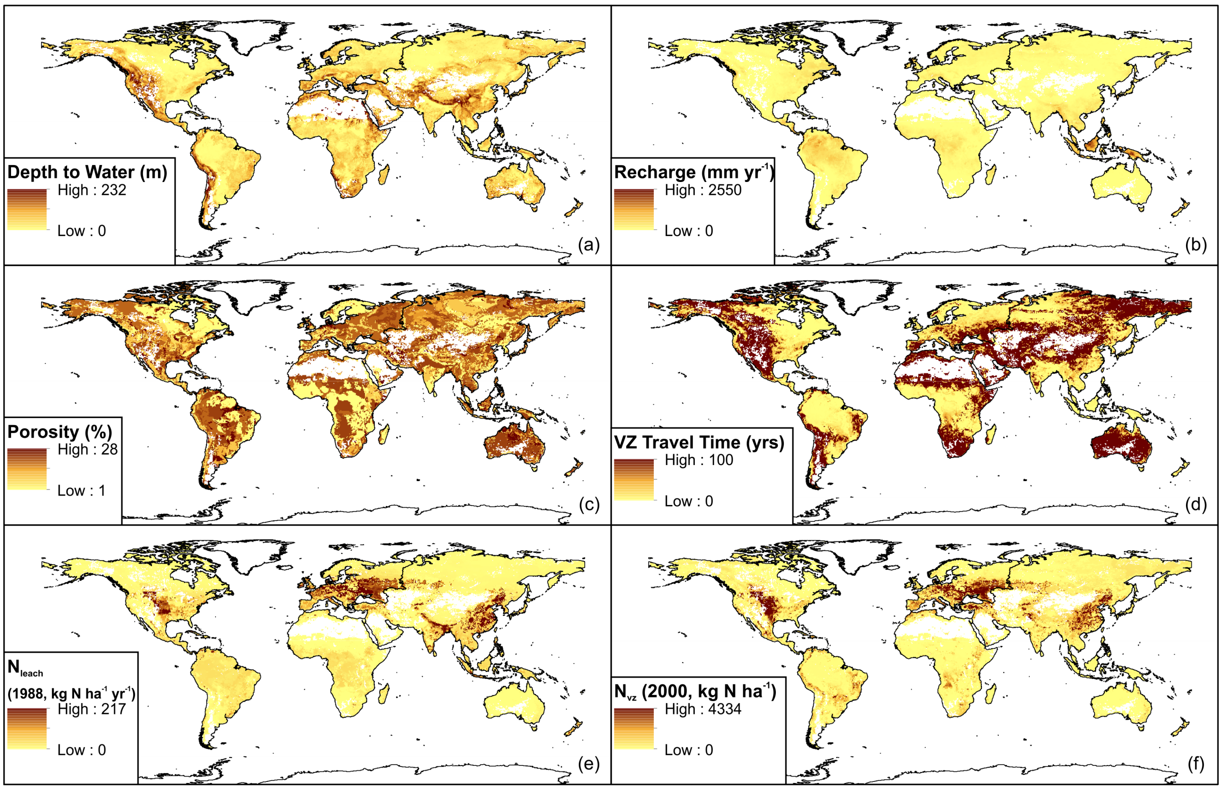

Supplementary Fig. 2: Global depth to water[2] (a), groundwater recharge[3] (b), porosity[4] (c) input datasets and derived vadose zone (VZ) travel times (d), nitrate leaching (N_{leach}) for 1988[5] (e), and vadose zone nitrate-N storage (N_{vz}) in 2000 (f)

Supplementary References

1. Van Drecht G, Bouwman AF, Knoop JM, Beusen AHW, Meinardi CR. Global modeling of the fate of nitrogen from point and nonpoint sources in soils, groundwater, and surface water. *Global Biogeochemical Cycles* **17**, (2003).

2. Fan Y, Li H, Miguez-Macho G. Global Patterns of Groundwater Table Depth. *Science* **339**, 940-943 (2013).

3. Wada Y, van Beek LP, van Kempen CM, Reckman JW, Vasak S, Bierkens MF. Global depletion of groundwater resources. *Geophysical Research Letters* **37**, (2010).

4. Gleeson T, Moosdorf N, Hartmann J, van Beek LPH. A glimpse beneath earth's surface: GLobal HYdrogeology MaPS (GLHYMPS) of permeability and porosity. *Geophysical Research Letters* **41**, 2014GL059856 (2014).

5. Beusen AHW, Van Beek LPH, Bouwman AF, Mogollón JM, Middelburg JJ. Coupling global models for hydrology and nutrient loading to simulate nitrogen and phosphorus retention in surface water – description of IMAGE-GNM and analysis of performance. *Geosci Model Dev Discuss* **8**, 7477-7539 (2015).
